# Measurement Duration but Not Distance, Angle, and Neighbour-Proximity Affects Precision in Enteric Methane Emissions when Using the Laser Methane Detector Technique in Lactating Dairy Cows

**DOI:** 10.3390/ani12101295

**Published:** 2022-05-18

**Authors:** Raphaël Boré, Thiphaine Bruder, Mohammed El Jabri, Margaret March, Paul R. Hargreaves, Benoît Rouillé, Richard J. Dewhurst, Mizeck G. G. Chagunda

**Affiliations:** 1 Institut de l’Élevage, 149 Rue de Bercy, 75012 Paris, France; raphael.bore@idele.fr (R.B.); mohammed.eljabri@idele.fr (M.E.J.); benoit.rouille@idele.fr (B.R.); 2 Nasekomo, 1612 Sofia, Bulgaria; thiphaine.bruder@gmail.com; 3 SRUC (Scotland’s Rural College), Kings Buildings, West Mains Road, Edinburgh EH9 3JG, UK; maggie.march@sruc.ac.uk (M.M.); paul.hargreaves@sruc.ac.uk (P.R.H.); richard.dewhurst@sruc.ac.uk (R.J.D.); 4 Department of Animal Breeding and Husbandry in the Tropics and Subtropics, University of Hohenheim, Garbenstr. 17, 70599 Stuttgart, Germany

**Keywords:** laser methane detector (LMD), enteric methane, measurement reliability

## Abstract

**Simple Summary:**

Methane that is breathed out and eructed from ruminants is a potent greenhouse gas that contributes to climate change. Although metabolic chambers are the “gold standard” for measuring methane from livestock, their application in production farms is very limited. There is a need to develop proxy methods that can be applied in such production environments. The proprietary Laser Methane Detector (LMD) has been trialed for the previous decade and has demonstrated its usefulness as a non-invasive and portable instrument to determine methane output from ruminants. In validating the reliability and stability of the data generated by the LMD, the current study gives answers to some very practical assumptions used in the use of the LMD and enhances the confidence in its use in ruminants.

**Abstract:**

The laser methane detector (LMD), is a proprietary hand-held open path laser measuring device. Its measurements are based on infrared absorption spectroscopy using a semiconductor laser as a collimated excitation source. In the current study, LMD measurements were carried out in two experiments using 20 and 71 lactating dairy cows in Spain and Scotland, respectively. The study aimed at testing four assumptions that may impact on the reliability and repeatability of the LMD measurements of ruminants. The study has verified that there is no difference in enteric methane measurements taken from a distance of 3 m than from those taken at a distance of 2 m; there was no effect to the measurements when the measurement angle was adjusted from 90° to 45°; that the presence of an adjacent animal had no effect on the methane measurements; and that measurements lasting up to 240 s are more precise than those taken for a shorter duration. The results indicate that angle, proximity to other animals, and distance had no effects and that measurements need to last a minimum of 240 s to maintain precision.

## 1. Introduction

The laser methane detector (LMD) is a proprietary hand-held open path laser measuring device. Its measurements are based on infrared absorption spectroscopy using a semiconductor laser as a collimated excitation source. It employs second harmonic detection of wavelength modulation spectroscopy to establish methane concentration [1]. The LMD is manufactured by Tokyo Gas Engineering Solutions, Ltd. (Tokyo, Japan) and was originally developed for the detection of gas leaks, and therefore, can discriminate between high CH_4_ concentrations and the low background concentration in the atmosphere [2]. Since its use in livestock methane determination was first introduced by Chagunda and co-workers [3], different versions of this device have been developed but using the same technology, namely tunable diode laser absorption spectroscopy. In dairy cows, different methods are used to measure methane. These include respiration calorimetry chambers, isotopic techniques, tracer techniques (sulphur hexafluoride (SF_6_)), and mass balance/micro-meteorological techniques [4].

Although the above-mentioned techniques are effective and efficient at a controlled experimental level, their application at a participatory and applied research level, which is usually carried out at commercial dairy farms, is very limited [3]. It is for this reason that we choose to work with the portable and easily accessible LMD. The most recent generation of the LMD, the Laser Methane Mini-Green^®^ (LMm-g^®^; Tokyo Gas Engineering Solutions, Ltd., Tokyo, Japan), was used to measure profiles of the CH4 concentration in ppm × m, the cumulative CH_4_ concentration along the laser path in meters, in the breath of cows. The principle of the measuring technology was described previously by Chagunda and co-workers. [3,5]. The LMm-g is connected to a smartphone or tablet running the GasViewer app (Tokyo Gas Engineering Solutions) via Bluetooth connection for exporting and storing the data. It uses tunable diode laser absorption spectroscopy [6]. The wavelength of the indium-gallium-arsenide laser (1653 nm) is specific for a strong absorption band of methane. The reflected laser beam is detected by the device, and its signal is processed and converted to the cumulative methane concentration along the laser path in parts per million-meter (ppm-m). With this it is assumed that the distance between the LMD and the animal should affect the concentration measured and should be corrected or accounted for. When an animal either breathes out or eructates, a plume is exhaled. As such, the methane concentration measured should ideally be affected by the size of the plume. Thus, it is important to investigate the effect of the measurement angle. Further, the presence of adjacent animals could disturb the concentration of methane measured if a plume exhaled by one animal is interfered by another animal. Although different studies have so far employed the LMD to measure methane in predominantly cattle, sheep, and goats, the measurement protocols have applied the underlaying assumptions that were proposed in the feasibility and earlier studies [3]. Three of these assumptions, although based on sound and fundamental biology, gas dynamics, and laser physics, had until now mostly only been partially systematically tested [3,7,8]. These three assumptions are those that may impact on the reliability and repeatability of the laser measurements. These assumptions are that (a) as long as the person taking methane measurements is within 3 m to the cow, the measurements are not significantly affected [3], (b) the plume effect of a neighboring methane point-source, in this case a cow, does not affect the methane measurements being taken (Tokyo Gas Engineering Solutions) [2], and (c) the angle at which the LMD is, relative to the methane point-source, does not have a significant effect on the methane measurements (Tokyo Gas Engineering Solutions) [2]. These three foregoing assumptions form the hypotheses that were tested in this study. Further, there has been a debate on the need for a common measurement protocol. On this, there are two schools of thought. On the one hand, the proponents of the common protocol urge that this will create an ease of comparison of results from different breeds, production systems, and environments. It would also help develop a robust set of emission factors associated with different ruminants through a common dataset. On the other hand, some researchers urge for a more flexible approach where researchers develop and define their own protocols that could be applied in their environment and practical situation. Whichever school of thought one would align with, there is a general consensus on the need for further research in developing either a joint protocol or a common guideline for enteric methane measurements and data analysis from ruminants [9]. The objectives of the current study were to examine the three main measuring assumptions of the LMD in a systematic and robust manner. Specifically, the study investigated the effect of distance between the animal and the LMD; the effect of the measurement angle; and the effect of the presence of an adjacent animal on the reliability of the LMD measurements. Further, the study tested different measurement durations in order to get good value of enteric methane data from the animals’ breath cycles. These objectives were important in order to identify the best combination of measurement situations to ensure repeatable and reliable measurements of enteric methane released by animals.

## 2. Materials and Methods

### 2.1. Data Collection

In order to address the first three objectives, a study involving 20 lactating dairy cows of different age, number of lactations, and milk production was conducted. The cows were fed a total mixed ration (TMR) established to cover the needs of dairy cows. The study was conducted at the EVAM dairy research facility (IRTA,) 17121 Monells, Girona (latitude 41.97 N and longitude 2.99 W) in Spain. Cows were housed in a standard free stall (cubicle) dairy barn. The Barn had open (slated) sides with veils that could be drawn down in very cold seasons and rolled up in the summer. Cubicles had rubber beds with sawdust bedding. The rest of the shed was made up of passageways, loafing, and feeding area. Animals were randomly chosen into the study that was conducted over a period of five weeks. During the five weeks, four different animals were housed in a separate pen within the same animal barn each week to facilitate measurements measurement. Animal group characteristics were detailed in the Table 1.

**Figure 1:**
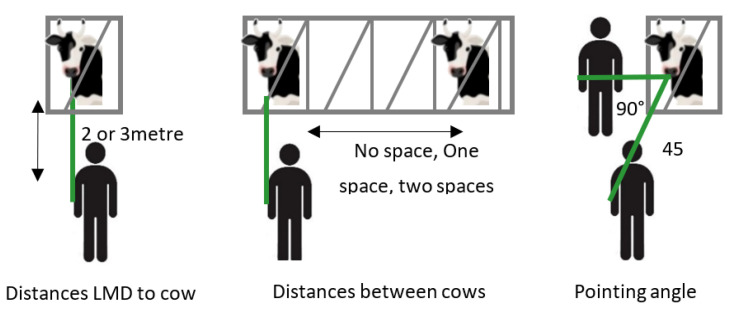
The measurement combinations that were applied during the systematic validation of measuring assumptions of the LMD as applied in dairy cattle.

Enteric methane measurements were obtained in four-minute measurement-windows on each cow over a period of 4 days for each cow. There were twelve measurement combinations that were applied. In order to collect enough data and with the requires statistical power to test all the three objectives in a systematic way, the following measurement combinations were carried out: two variants of the distance between the device and the nostril of the cow (2 and 3 m); two variants of the monitoring angle (45° and 90°); and three variants of the distance between adjacent animals (0 gap, 1 animal width, 2 animal width between animals). The measurement combinations are illustrated in Figure 1.

Measurements were repeated 3 times on the same day to account for any potential effect on methane emissions due to rumen fill. In practice, this translates to 3 different combinations of measurements having been run 3 times on 4 animals each day. Each combination was randomly selected for each animal per day and per measurement time (during morning feeding approximately 07:00 h, midday approximately 12:00 noon and after evening milking approximately 18:00 h). As such the four animals for each measurement time were chosen randomly. In total, 720 measurements (36 measurements from each animal) of 4 min were spread out over 20 days.

### 2.2. Enteric Methane Phenotypes

The profile of breath cycles representing both respiration and eructation were plotted from the data. Such profiles were used for visual inspection as data quality control procedures before any further processing. The height of peaks during an eructation event is higher than the height of peaks during respiration. In defining the phenotypes, respiratory and eructation CH_4_ values for each profile were separated using a threshold value. Standard deviation for each profile was used as the threshold value separating respiration and eructation as proposed in previous studies [5]. All methane values that were a standard deviation above the mean were considered as eructation and respiration. From this separation a phenotype P_MEAN was calculated as the arithmetic mean of all peak CH4 values as defined in a previous study [10]. This procedure was implemented through an automated algorithm that detects peaks in the CH_4_ profile in two steps. In step one the difference between a data point (x) and the preceding value (x − 1) in the time series of one profile was calculated. In step two, if the difference between x and xi−1 was less than zero, and the difference between the two data points (x − 1) and (x − 2) were more than zero, then the data point (x − 1) was classified as a peak. Values of all P_MEAN values in any profile were calculated generating one-point measurement per profile.

A separate trial was carried out to address Objective 4 of this study, which was to test whether a measurement length of 4 min was sufficient to capture a number of complete breath cycles and an adequate amount of episodes of eructation. The main research questions were; would shorter measurement durations give less information? Would longer measurement lengths provide more information? As with the questions in the first three objectives, addressing these questions would assist in finding an optimal measurement time in order to provide a set of practical utilization rules for the LMD. A summary of research questions posed regarding measurement length are presented in Table 2.

To address this objective data from a group of 71 dairy cows, which were in a 5 week-experiment at Scotland’s Rural College Dairy Research Centre, Dumfries, Scotland (latitude, 55°04′ N and longitude 3°37′ W) were used. Each week for 3 days, measurements were taken from 15 different cows after midday milking. The distance between the laser beam and the nostril of the cow was estimated at 1 m. Measurements lasted a duration of 4 to 5 min. For each individual time-series measurement, the cow’s tag number and time of recording were documented. The mean of the peak methane measurement (P_MEAN) of these 71 cows was 196.44 (sd = 40.35) ppm-m.

### 2.3. Data Analysis

Descriptive statistics, mean, standard deviation, maximum, and minimum values were calculated to determine the distribution of the data. To determine the effect of distance between the animal and the LMD, the effect of the measurement angle, and the effect of the presence of an adjacent animal on the reliability of the LMD measurements, an analysis of variance was used. The analysis of variance applied a mixed model that accounted for all explanatory variables that were to be tested and covariates. The covariates that were included in the mixed model were cow, recorder, and time of day. Both classic and Bayesian approaches were used in the analysis.

To test and compare 4 min of measurement, five different recording windows of 60, 120, 180, 240, and 300 s were created. For each of these windows, a methane gross average was calculated. For the repeated measures and mixed models with “Day” nested within “CowID”, dates of measure are not the same depending on the cow. The Model was written:

Y_ijkl_ = µ + α_i_ + τ_j_ + β_jk_ + ε_ijkl_

with:

− Y_ijkl_, average value of the α_i_ measurement time, the τ_j_ cow, the β_jk_ date, and the lth sample;
− µ, α_i_, τ_j_ remain the same;
− β_jk_, nested factor with “CowID” i. β_jk_ effects follow a Normal Distribution with a variance σ^2^_CowID:Day_;
− ε_ijk_, independent residuals N(0, σ^2^).

To compare the different Measurement Times, a pair-wise comparison was carried out. For each comparison, a hypothesis that estimators were significantly different was either accepted or rejected or not (*p* ≤ 0.05).

## 3. Results

**Figure 2:**
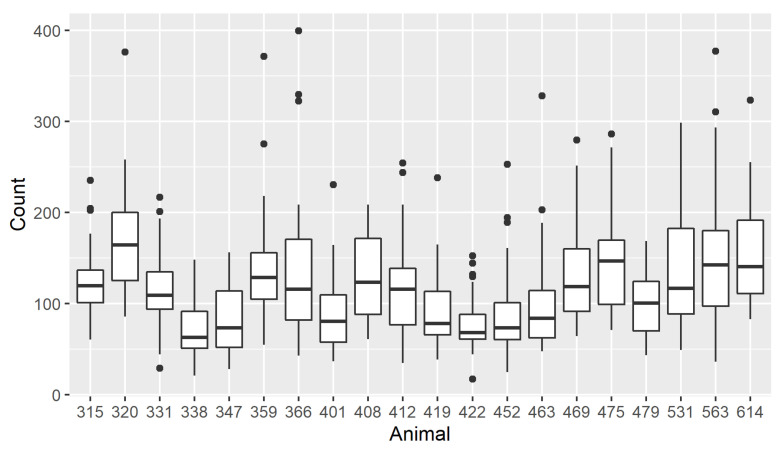
Distribution of P_MEAN (ppm-m) by animal.

**Figure 3:**
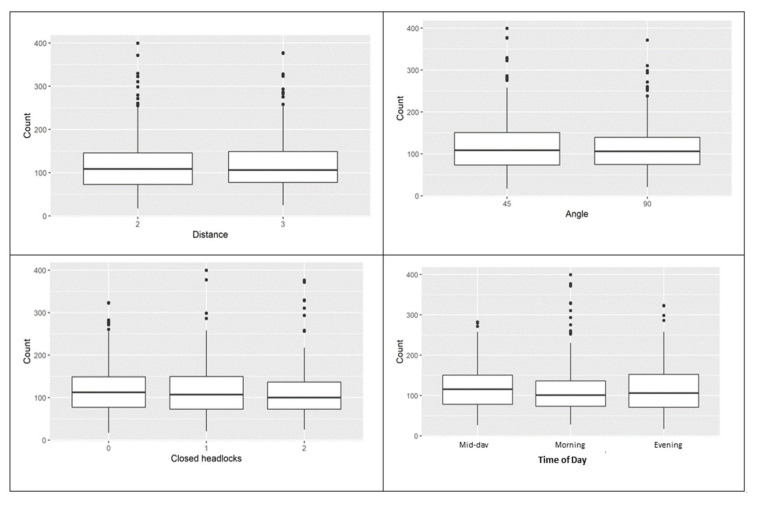
Distribution of P_MEAN (ppm-m) by distance (**top left**), by angle (**top right**), by closed headlock (**bottom left**), and by time of day (**bottom right**).

The distribution of P_MEAN (ppm-m) by animal and by treatment groups are presented in Figure 2 and Figure 3, respectively.

In the individual cow distribution, there was a marked difference among individuals. This reflects the individual variation arising from the biology of the individual animals. However, group averages did not exhibit marked differences. The descriptive statistics for the individual cows in the study that addressed the first three objectives are presented in Table 3.

The phenotype P_MEAN ranged from 17.2 to 399.5 ppm-m. The average measurements for methane across all the tested factors were in the same range. However, an examination of the averages within each factor showed numeric differences in the variation around the mean. This variation around the mean was quantified by dividing the standard deviation by the average values that yielded the statistic, coefficient of variation (CV%). Within the factor “distance”, methane measurements from a 2 m distance had a slightly higher cv% than measurements from a 3 m distance (cv% = 51.1% vs. 49.5%). Similar numerical differences were observed in measurement angle, headlock, and time of day. Measurements conducted from the right angle had numerically lower variation around them than those taken from a 45° angle (cv% = 47.6% vs. 52.4%). In the times when there was no space between two cows, there was lower variation than when some space was left between cows (cv% = 47.8% for one space vs. 51.8% and 51.2% for a space and two spaces, respectively). Methane measurements taken in the morning had a numerically higher variation than those taken during the afternoon and the evening (cv% = 55.7% for morning, 44.6% for afternoon, and 50.3% for evening).

An analysis of variance applying a mixed model that accounted for all explanatory variables that were tested and covariates generated no significant effects of any of the effects that were tested. In all cases, the only variable that had a significant effect was the individual cow effect. This indicated the differences coming from the individual biology of the cow while rejecting the hypothesis that distance, measurement angle, proximity to another cow, and time of day would significantly affect methane measurements from a cow when using the LMD. Results from the fourth objective indicated that methane measurements taken over a duration of 240 s were significantly different from 60 s (*p* < 0.001) and numerically different from those taken in 120 s (Table 4).

## 4. Discussion

The current study aimed to validate the four main measuring assumptions of the LMD in a systematic and robust manner. Since the LMD was not originally developed for enteric methane measurements in ruminants, the validation of these assumptions revolves around two questions. First, on the properties and speed of response of the laser beam during an encounter with methane concentrations in eructations and breath. Second, the bio-dynamic properties of the methane plume generated from eructations that may either be a single eructation peak or a series of peaks with decreasing methane concentrations as an eructation gets drawn in and out of the lungs.

On the question of on the properties and speed of the response of the laser beam during an encounter with methane concentrations in eructations and breath, the laser (light amplification by stimulated emission of radiation) is a coherent and amplified beam of electro-magnetic radiation [11]. The key element in making a practical laser is the light amplification achieved by stimulated emission due to the incident photons of high energy. A laser comprises three principal components, namely, the lasing medium, means of exciting the lasing medium into its amplifying state (lasing energy source), and its optical delivery/feedback system [11]. Having photons of the same frequency, wavelength, and phase, makes laser light different from ordinary light and hence can be used to measure different elements with high level of specificity. Thus, unlike ordinary light, laser beams are highly directional, have a high-power density, and better focusing characteristics [11]. It is these principal components and characteristics that are applied in the LMD.

The four objectives in the current study principally examined two phenomena that deal with the loss of spatial coherence of an initially coherent wave due to propagation through a random medium [12]. These phenomena, which have important consequences on the behaviour of that wave at the receiver, are the angle-of-arrival and beam wander. It is well known that atmospheric turbulence causes significant variations in the arrival angle of laser beams used in free-space communications [12]. However, laser beams and the distances used in measuring methane from ruminants are far too dismal to register this effect. For example, previous studies [13] have indicated that a ray tracing approach implemented to examine the chromatic divergence and angle-of-arrival of the rays projected over a distance of 150 km along the ground through various practical and extreme atmospheric conditions involving a temperature inversion layer, caused pairs of rays with wavelengths 532 and 1550 nm to diverge up to 4.5 times greater than their standard atmosphere predictions. The other phenomenon that would have been hypothesised is that of beam wander. Beam wander is a random deflection caused by large-scale inhomogeneities of the atmospheric turbulence that a finite optical beam experiences as it propagates [14]. Because bean wander is caused mostly by large-scale turbulence, diffraction effects are often negligible [14]. Again, confirming the non-significant results obtained in the current study.

On the question of the bio-dynamic properties of the methane plume, when interpreting the effects of distance from the nostril, or angles at which LMD measurements are taken, the ideal situation would be to know the size and shape of the breath plume, as well as the way that it expands and mixes with ambient air. Most rumen gasses are taken into the lungs before being expired, so we would never record methane concentrations found in pure rumen headspace gas (approx. 25%;) [15]. Simplifying to consider breath plumes as spheres, we can consider the tidal volume from a dairy cow of between 3.1 to 4.4 L as a sphere with diameter 20.4 cm [16,17]. This would expand 940-fold to fill a sphere of 2 m diameter and 3200-fold to fill a sphere of 3 m diameter. The implication of this is that the concentration of methane between 2- and 3-metres away from the nostril will be 30% of that with 2 m of the nostril (i.e., [((2 m × 1) + (1 m × 0.3))/3 m]). In fact, the LMD only measures the concentration of methane within the detectable methane plume, with results expressed in ppm-m, that is methane concentration (ppm) multiplied by the thickness (m) of the measured plume (Tokyo Gas Engineering Solutions Corporation, 2022) [18]. Since our study showed no difference in methane (ppm-m) between measurements taken at a 2 m distance and a 3 m distance, it seems likely that any dilution effect in methane concentration at this distance has a minimal effect. In the current study, no measurements were taken at a 1 m distance. At the time of designing the experiment, the general consensus was to test those distances that have not been used in other studies, and hence 2 m and 3 m were used. Although this should not have affected the results presented herein, future studies should include many more distances in order to generate a full profile.

In general, compared with previous studies, the minimum for P_MEAN in all subcategories of experiment one were lower (17–28 ppm-m), e.g., [7,8]. Although this could have no direct effect on the results in the current study, because of this being a point measurement derived from a 4-min recording, at least one eructation event should have been recorded. These low minimum values may have been due to either the genetics of the cows involved or the physiological nature of the concentration of methane in their breath as opposed to eructations [15].

The current study did not investigate the conversion of enteric methane concentration from spot measurements into methane volumes per day. It is for that reason that no control with a different technique such as sf6 or greenfeed unit was applied. Ancillary studies will address this issue and also include more animals in the study because the advantages of using the LMD as shown by different researchers [8], highlights the usefulness of the non-invasive and portable instrument to determine farm methane output.

## 5. Conclusions

The study has verified that (1) there is no difference in enteric methane measurements taken from a distance of 3 m from those taken from 2 m; (2) there was no effect to the measurements when the measurement angle was adjusted from 90° to 45°; (3) that the presence of an adjacent animal had no effect on the methane measurements; and (4) that measurements lasting up to 240 s are better than those taken on a shorter duration. In validating the reliability and stability of the data generated by the LMD, the current study gives answers to some very practical questions and enhances the confidence in the use of the LMD in ruminants and hence strengthens the case for a unified measurement protocol.

## Figures and Tables

**Table 1:** Animal characteristics and methane measurement dates for the group of cows involved in the methane measurements study.

Characteristics *	Group 1	Group 2	Group 3	Group 4	Group 5
First Day of LMD	9 February 2021	16 February 2021	23 February 2021	2 March 2021	9 March 2021
Last Day of LMD	12 February 2021	19 February 2021	26 February 2021	5 March 2021	12 March 2021
Number of cows	4	4	4	4	4
Lactation rank	4.00	3.00	3.25	2.50	3.50
DIM (d)	114	130	166	153	146
DMI in 8 previous days (kg/d)	20.2	21.9	22.8	26.5	24.6
Milk Yield (kg/d)	26.2	29.5	29.2	36.2	30.0
Body Weight (kg)	833	793	739	742	687

* LMD = Laser methane Detector; DIM = days in milk; DMI = dry matter intake.

**Table 2:** Research questions on the enteric methane measurement duration for ruminants.

Compared to a 240 s Measurement	Question
At 60, 120, and 180 s	Is information lost at shorter durations? Is a 240 s measurement necessary?
At 300 s	Is information added at longer durations? Is a 240 s measurement satisfactory?

**Table 3:** Descriptive statistics of LMD data by factors and modalities. Statistical analysis of P_MEAN.

Factor	Modality	N	Min	Max	Mean	Median	sd	Iqr
Distance	2	360	17.2	399.5	116.9	108.3	59.8	72.9
Distance	3	360	24.5	377.3	118.0	106.1	58.4	71.8
Angle	45	360	17.2	399.5	120.3	108.5	63.1	77.7
Angle	90	360	20.7	371.7	114.7	106.0	54.6	65.1
Closed headlock	0	240	17.2	323.2	120.5	112.2	57.6	72.1
Closed headlock	1	240	20.7	399.5	119.0	106.9	61.6	77.0
Closed headlock	2	240	24.5	376.1	113.0	99.9	57.9	64.0
Time of day	Morning	240	28.0	399.5	117.1	100.8	65.2	63.1
Time of day	Afternoon	240	26.6	282.5	119.5	115.7	53.3	72.5
Time of day	Evening	240	17.2	323.2	115.8	106.3	58.2	81.5

**Table 4:** Least square means of enteric methane measurements calculated from different measurement durations *.

Duration (s)	Lsmean (ppm-m)	Std Error
60	135.78 ^a^	1.54
120	143.42 ^b^	1.54
180	145.32 ^b^	1.53
240	148.08 ^b^	3.06

* Number of cows = 71; Number of observations = 1075; Different superscripts on lsmeans indicate significant difference *p* < 0.001 line 246.

## Data Availability

The data presented in this study are available on request from the corresponding author.
